# Serpinc1 Acts as a Tumor Suppressor in Hepatocellular Carcinoma Through Inducing Apoptosis and Blocking Macrophage Polarization in an Ubiquitin-Proteasome Manner

**DOI:** 10.3389/fonc.2021.738607

**Published:** 2021-11-22

**Authors:** Dacai Xu, Jiawen Wu, Liang Dong, Wenwen Luo, Lanying Li, Daolin Tang, Jinbao Liu

**Affiliations:** ^1^ Guangzhou Institute of Pediatrics, Guangzhou Women and Children’s Medical Center, Guangzhou Medical University, Guangzhou, China; ^2^ Institute Pasteur of Shanghai, Chinese Academy of Science, Shanghai, China; ^3^ Affiliated Cancer Hospital and Institute of Guangzhou Medical University, Guangzhou Municipal and Guangdong Provincial Key Laboratory of Protein Modification and Degradation, School of Basic Medical Sciences, Guangzhou Medical University, Guangzhou, China; ^4^ Department of Surgery, University of Texas (UT) Southwestern Medical Center, Dallas, TX, United States

**Keywords:** serpinc1, apoptosis, liver cancer, macrophage, ubiquitination

## Abstract

Serpinc1 is a serine protease inhibitor in the coagulation cascade, but its role in tumor biology remains obscure. Here, we report an unexpected role of serpinc1 in suppression of hepatocellular carcinoma (HCC). In HCC patients, the mRNA and protein expression of serpinc1 is upregulated, which is negatively correlated with tumor grade, and has a better prognosis than patients with low serpinc1. In addition, patients with high expression of serpinc1 generally have a better tumor immune microenvironment, accompanied by changes in multiple immune cells and mediators. In particular, tumor-promoting M2 macrophages are negatively correlated with serpinc1 expression and the prognosis of HCC patients. *In vitro* experiments further show that overexpression of serpinc1 inhibits the growth of HCC cells (HepG2 and SMMC7721) by inducing apoptosis. Accordingly, cell co-culture experiments reveal the direct role of serpinc1-overexpressed HCC cells in inhibiting the formation of M2 macrophages. Subsequent unbiased quantitative proteomic and ubiquitinome analyses identify that multiple poly-ubiquitination of proteins involved in signal pathways (such as autophagy, apoptosis, lactate metabolism, and VEGF signaling) are regulated by serpinc1. Overall, these findings establish a serpinc1-dependent ubiquitin-proteasome system to control apoptosis and antitumor immunity.

## Introduction

Hepatocellular carcinoma (HCC) is a primary malignant tumor of the liver and the leading cause of tumor-related mortality worldwide (1, 2). The estimated number of new cases ranks sixth among all cancers, while the estimated number of deaths ranks fourth (3–5). More than 800,000 people are diagnosed with this cancer each year throughout the world. The incidence of HCC is highest in Asia, especially in China. China’s liver cancer death rate jumped from the third highest cancer death rate in 2018 to the second highest in 2020 (6, 7). The potent metastasis and invasion of HCC primarily impact the prognosis and recurrence of HCC (8, 9). Therefore, it is urgent to find new targets for HCC treatment.

The serine peptidase inhibitor clade C member 1 (serpinc1) is a serine protease inhibitor that regulates coagulation balance (10). The HUMAN PROTEIN ATLAS analysis suggests that serpinc1 mRNA is specially expressed in normal liver and gallbladder tissues, rather than other tissues. However, serpinc1 mRNA transcription can be detected in central nervous system lymphoma, and high serpinc1 mRNA expression predicts a poor prognosis (11). Serpinc1 could inhibit tumor migration, invasion, and angiogenesis in certain cancers (12, 13). Although serpinc1 knockdown inhibits the growth of nasopharyngeal carcinoma (14), the depletion of serpinc1 favors liver tumorigenesis induced by diethylnitrosamine and CCl_4_ treatments in mice through neutrophil/IL-8 signaling (15, 16). However, serpinc1 is upregulated in HCC compared to normal controls (17). Moreover, serum serpinc1 was reported to be a valuable marker for the prognosis of HCC patients undergoing curative hepatectomy (18, 19). These findings indicate the context-dependent role of serpinc1 in the tumor microenvironment.

Cancer immunotherapy is a powerful cancer treatment method that relies on the communication between tumor cells and immune cells. This is a multistep process, including the release of cancer antigens (step 1), presentation of cancer antigens by dendritic cells or other antigen presenting cells (step 2), priming and activating T cells and antigen-presenting cells (step 3), transportation of T cells to cancer (step 4), infiltration of T cells into cancer (step 5), recognition of cancer cells by T cells (step 6), and finally killing cancer cells (step 7) (20). The polarization of immune cells can fine-tune the effects of immunotherapy. In particular, the polarization of macrophages from type M1 to type M2 can lead to immune escape of tumor cells. Although macrophage M2 polarization can be target for the treatment of HCC (21–23), the mechanism of how to regulate macrophage M2 polarization is poorly understood.

In this study, we report that serpinc1 is a key regulator of HCC tumor immunity, which induces apoptosis in cancer cells, and ultimately limits the polarization of macrophage M2 through the ubiquitin-proteasome system (UPS). Importantly, the increased serpinc1 expression significantly improves the prognosis of HCC patients with activated antitumor immune microenvironment.

## Materials and Methods

### Cell Culture

HepG2, SMMC7721, and THP1 cell lines were purchased from the American Type Culture Collection (Manassas, VA, USA). HepG2, SMMC7721, and THP1 cells were grown in RPMI 1640 medium (Gibco Invitrogen, Carlsbad, CA, USA) supplemented with 10% fetal bovine serum (FBS, purchased from Bioworld Technology, Louis Park, MN, USA).

### Transfection and Transduction

The plasmid FLAG-serpinc1 encoded a fusion protein of serpinc1 and FLAG. The FLAG-serpinc1 plasmids were linked in pcDNA3.1 (+). The serpinc1 overexpression lentivirus was linked in plent-EF1a-FH-CMV-GFP-P2A-puro vector. The serpinc1 shRNA lentivirus was constructed in pLent-U6-GFP-Puro vector. The sequences of serpinc1 shRNA were listed as below: shRNA-1: GAT CCG CCG AAT CAC CGA TGT CAT TCT TCA AGA GAG AAT GAC ATC GGT GAT TCG GCT TTT TTA; shRNA-2: GAT CCG GAA GGA ACT GTT CTA CAA GGT TCA AGA GAC CTT GTA GAA CAG TTC CTT CCT TTT TTA; shRNA-3: GAT CCG GCA TTT CTT GAG GTA AAT GAT TCA AGA GAT CAT TTA CCT CAA GAA ATG CCT TTT TTA. All the plasmids and lentivirus were purchased from VigeneBio (Shandong, China). For FLAG-serpinc1plasmid transfection, HepG2 or SMMC7721 cells were randomly seeded in 60 mm dishes for 24 h and then transfected with FLAG-serpinc1plasmid in RPMI opti-MEM medium using Lipofectamine 2000 reagent (Life Technologies, Invitrogen, CA, USA) according to the manufacturer’s instructions. After 6 h of transfection, the fresh medium was changed, and the cells were cultured for 48 h for further analysis. For lentivirus transduction, HepG2 or SMMC7721 cells were randomly seeded in 60 mm dishes for 24 h, and then transducted with serpinc1 shRNA or overexpression lentivirus (MOI=100). The fresh medium was replaced 12 h after transfection, and the cells were harvested 48 h after transduction for further analysis. One µg/ml puromycin was used to select stable expression cells.

### Cell Viability

HepG2 or SMMC7721 cells were randomly seeded in 96-well plates at 8,000 cells per well for 24 h. Cells were transfected with control or FLAG-serpinc1plasmid for 48 h. The CellTiter 96^®^ AQueous One Solution Cell Proliferation Assay (MTS) (Promega, Shanghai, China) was added to wells 3 h before termination of assays according to the manufacturer’s instructions. The absorbance at 490 nm was estimated using a plate reader (Varioskan Flash 3001, Thermo, Waltham, MA, USA).

### Western Blot

Briefly, cell lysates were separated on sodium dodecyl sulfate (SDS)-polyacrylamide gel and then transferred to PVDF membrane (Millipore, Bedford, MA, USA). The membrane was blocked with a PBS solution containing 5% non-fat dry milk and 0.1% Tween-20 for 1 h at room temperature, and then incubated with a primary antibody overnight at 4°C. Membranes were incubated with a horseradish peroxidase-conjugated secondary antibody in blocking buffer for 1 h, and then visualized using an ECL kit (Santa Cruz Biotechnology) and detected by exposing to X-ray films (Kodak, Rochester, NY, USA) or Imagelab system (Bio-rad). Anti-FLAG (14793) antibody was purchased from Cell signaling technology (Beverly, MA, USA). Anti-serpinc1 (ab126598) antibody was obtained from Abcam (Cambridge, UK). Anti-ubiquitin (sc-8017) antibody was purchased from Santa Cruz Biotechnology (Santa Cruz, CA, USA). Anti- GAPDH (AP0063) antibody was obtained from Bioworld technology (Louis Park, MN, USA). Anti-BCL-XL (2764), MCL-1 (5453), BCL2 (4223), BAX (5023) antibodies were purchased from Cell Signaling Technology (Shanghai, China).

For immunoprecipitation, HepG2 cells stably overexpressed control or serpinc1 were seeded in dishes. One µM bortezomib (selleck chemicals, S1013, Shanghai, China) was added into culture medium 4 h before harvesting the cells. The cells were lysed with RIPA buffer containing protease inhibitors and 50 µM PR-619 (selleck chemicals, S7130, Shaghai, China). After adjusting the concentrations, lysates were incubated with HIF1A (abcam, ab51608) or HMGB1 (abcam, ab228624) antibody (dilution 1:200) in a shaker for 30 min at room temperature, and mixed with protein A/G magnetic beads (Bimake, B23201, Shanghai, China) overnight in a shaker at 4°C. Finally, proteins were eluted and denatured for Western blotting.

### Flow Cytometry

Trypsinized cells were washed and resuspended in PBS supplemented with 2% FBS. To examine apoptotic status, cells were stained with AnnexinV-FITC/PI or AnnexinV-PE/7-AAD (KeyGENBioTECH, Nanjing, China). For cell cycle detection, cells were fixed by 70% ethyl alcohol (v/v) overnight at 4˚C, and cell cycle analysis was performed using a Cell Cycle Kit (BestBio, Shanghai, China). Apoptosis and cell cycle were performed by FACSCalibur flow cytometer (BD Biosciences, Franklin Lakes, NJ, USA). The results were analyzed using ModFit LT software (version 3.2; Verity Software House, Topsham, ME, USA). For macrophage polarization assay, 200 μl FACS buffer (2% FBS, 0.02% NaN_3_, and 2 mM EDTA in 1×PBS) was used to resuspend cells, and then cells were plated to 96-well plates for centrifugation (2,000 rpm, 1 min). CD163-BV421or CD80-PE antibody was incubated with the cells at 4°C for 10 min, and then the cells were washed once before analysis by flow cytometry using FACScanto (BD Biosciences, Franklin Lakes, NJ, USA). The data were processed and analyzed through FlowJo v7.6 (Ashland, OR, USA).

### Macrophage Polarization Detection

THP1 cells were plated in a six-well plate at 0.1×10^6^/well. THP1 cells were activated by 50 nM PMA (phorbol 12-myristate 13-acetate) for 24 h, and then the medium was refreshed. Activated THP1 cells were co-cultured with 0.1×10^6^/well HepG2 cells (overexpressed control or serpinc1 or alone) for 48 h. The cells were collected to detect macrophage M2 marker using CD163-BV421 antibody (Biolegend, Cat: 333612, San Diego, CA, USA) and M1 marker using CD80-PE antibody (eBioscience, 2203557, Shanghai, China) by flow cytometry.

### ELISA

HepG2 cells stably overexpressed control or serpinc1 were seeded in six-well plates (0.2×10^6^/well) for 24 h. Then, cells were washed and refreshed with 1 ml culture medium for 48 h. After centrifugation at 15,000 g for 10 min, the culture medium was collected and the ELISA kit was used to detect cytokines [including IL4 (Biolegend, 430301, San Diego, CA, USA), IL10 (Biolegend, 430601), and IL13 (Biolegend, 435207)] or lactate (BioVision, K607-100, Milpitas, CA, USA) according to the manufacturer’s protocols. The absorbance at 570 nm subtracted to 450 nm was estimated using a microplate reader (Multiskan GO, Thermo, Waltham, MA, USA).

### Immunohistochemistry

The collection of hepatocellular carcinoma samples, paraffin embedding, serpinc1 antibody staining, and pathological analysis were completed by Shanghai Outdo Biotech Company (151 Libing Road, Shanghai, China, 201213). All participants were recruited in accordance with the company’s agreement and ethical standards. Paraffin-embedded sections were stained with anti-serpinc1 antibody (Abcam, ab126598, Cambridge, UK). The pathologist analyzed the positive staining and staining scores of cancer cells and normal tissues around the tumor. Serpinc1 expression was calculated by multiplying the percentage of positives in each sample.

### Label-Free LC-MS/MS Analysis

The label-free LC-MS/MS analysis was performed by Jingjie PTM Bio Co.Ltd (Hangzhou, China). Samples were lysed and sonicated in lysis buffer (8 M urea, 1% Protease Inhibitor Cocktail, and 50 μM PR-619). And then samples were determined and adjusted concentrations. For digestion, the protein solution was reduced with 5 mM dithiothreitol for 30 min at 56°C, alkylated with 11 mM iodoacetamide for 15 min at room temperature in darkness, and then diluted to final urea concentration less than 2 M with 200 mM TEAB. Trypsin was added at 1:50 trypsin-to-protein mass ratio for the overnight and 1:100 for another 4 h digestion. To enrich modified peptides, tryptic peptides were resuspended in NETN buffer (100 mM NaCl, 1 mM EDTA, 50 mM Tris-HCl, 0.5% NP-40, pH 8.0) and incubated with prewashed anti-ubiquitin antibody beads (Lot number PTM-1104, PTM Bio) at 4°C overnight with gentle shaking. The bound peptides were eluted from the beads with 0.1% trifluoroacetic acid after washing four times with NETN buffer and twice with H_2_O. The eluted fractions were vacuum-dried.

For LC-MS/MS analysis, the resulting peptides were desalted with C18 ZipTips (Millipore) and dissolved in solvent A containing 0.1% formic acid and 2% acetonitrile in water, directly loaded onto a home-made reversed-phase analytical column with 25-cm length, 75 μm i.d. Peptides were separated at a constant flow rate of 500 nl/min on an EASY-nLC 1200 UPLC system with a gradient from 9 to 25% solvent B (0.1% formic acid in 90% acetonitrile) over 36 min, 25 to 35% in 18 min and climbing to 80% in 3 min, then holding at 80% for the last 3 min. The peptides were subjected to NSI source followed by tandem mass spectrometry (MS/MS) in Q ExactiveTM HF-X (Thermo) coupled online to the UPLC. The electrospray voltage applied was 2.2 kV. The m/z scan range was 350 to 1,600 for full scan, and intact peptides were detected in the Orbitrap at a resolution of 120,000. Peptides were then selected for MS/MS using NCE setting as 28, and the fragments were detected in the Orbitrap at a resolution of 15,000. A data-dependent procedure that alternated between one MS scan followed by 20 MS/MS scans with 15.0 s dynamic exclusion. Automatic gain control (AGC) was set at 5E4 ions/s. Fixed first mass was set as 100 m/z.

For Database Search, the resulting MS/MS data were analyzed using MaxQuant search engine (v1.6.15.0). Tandem mass spectra were searched against the Homo_sapiens_9606_SP_20201214.fasta (20395 sequences) concatenated with reverse decoy database. Trypsin/P was specified as cleavage enzyme allowing up to two missing cleavages. The minimum length of peptide segment was set to seven amino acid residues, and the maximum modification number of peptide segment was set to 5. The mass tolerance for precursor ions was set as 20 ppm in First search and 4.5 ppm in Main search, and the mass tolerance for fragment ions was set as 0.02 Da. Carbamidomethyl on Cys was specified as fixed modification, and acetylation on protein N-terminal, oxidation on Met, and ubiqutin on Lys were specified as variable modifications. The quantitative method was label-free, and FDR of protein identification and PSM identification was adjusted to <1%.

The mass spectrometry proteomics data have been deposited to the ProteomeXchange Consortium *via* the PRIDE (24) partner repository with the dataset identifier PXD026782.

### Statistical Analysis

To determine statistical probabilities, Student’s t test and one-way ANOVA were used where appropriate. Statistical analysis was performed by GraphPad Prism5.0 software (GraphPad Software). A P value of <0.05 was considered statistically significant.

## Results

### Serpinc1 Is Upregulated in HCC Patients

**Figure 1:**
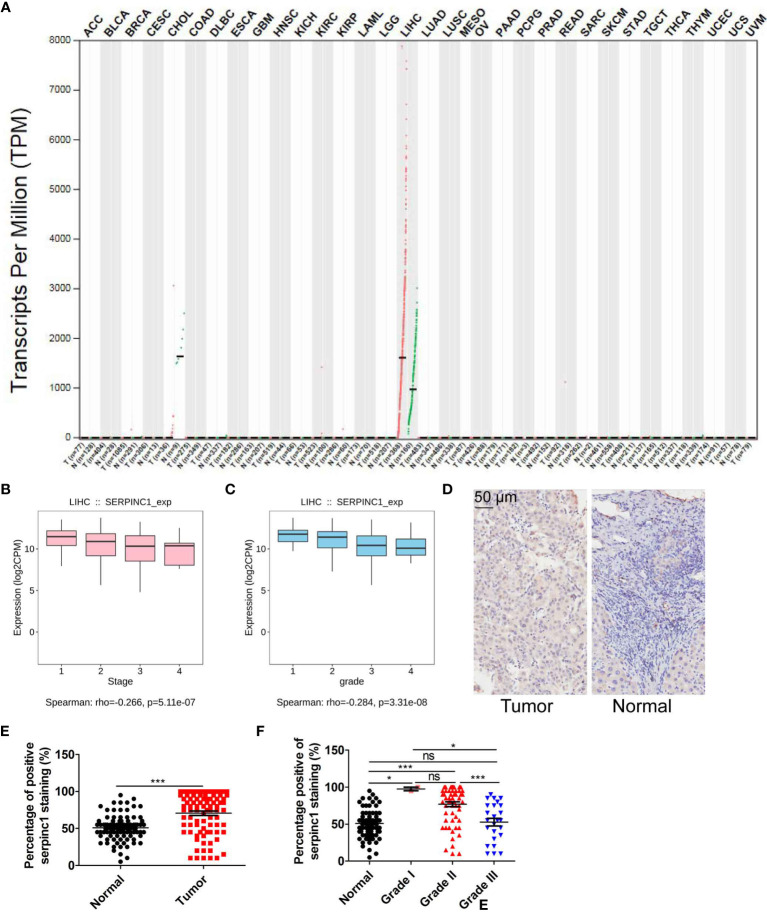
Serpinc1 is increased in hepatocellular carcinoma. **(A)** Analysis of the serpinc1 mRNA expression across cancers from GEPIA database. **(B, C)** TISIDB database to analyze the correlation of serpinc1 mRNA expression with individual tumor stages **(B)** and tumor grade **(C)**. **(D–F)** Hepatocellular carcinoma (n=90) and normal tissues (n=90) were stained with serpinc1 antibody. Representative images **(D)** and statistical analysis the percentage of positive serpinc1 staining in normal and tumor groups **(E)** or different grade groups **(F)**. *P < 0.05, ***P < 0.001, ns, no significant, *P* > 0.05.

Since serpinc1 plays different roles in liver tumorigenesis and nasopharyngeal carcinoma (14, 15), we first used the GEPIA database (http://gepia.cancer-pku.cn/index.html) to verify the effect of serpinc1 on 33 cancers (25). It is worth noting that compared with normal control tissues, serpinc1 mRNA levels were significantly increased only in tumor samples of HCC (**Figure 1A**), supporting the previous findings that serpinc1 has a potential role in HCC (17). We further analyzed the relationship between tumor grade/stage and serpinc1 mRNA expression in HCC using the TISIDB database (http://cis.hku.hk/TISIDB/) (26). This assay showed that serpinc1 mRNA levels were decreased both from grade 1 to grade 4 and stage 1 to stage 4 (**Figures 1B, C**). To support these bioinformatics analyses of serpinc1 mRNA expression, we performed immunohistochemical staining of serpinc1 in 90 pairs of HCC and normal tissues. In fact, the percentage of serpinc1 staining positive in HCC was higher than that of normal controls (**Figures 1D, E**). Further analysis of the relationship between serpinc1 protein expression and tumor grades revealed that the positive rate of serpinc1 staining was negatively correlated with tumor grades, which was consistent with the mRNA data from TISIDB database (**Figure 1F**). Collectively, these data suggested that serpinc1 is upregulated in patients with HCC.

### Serpinc1 Affects the Outcome of HCC Patients

**Figure 2:**
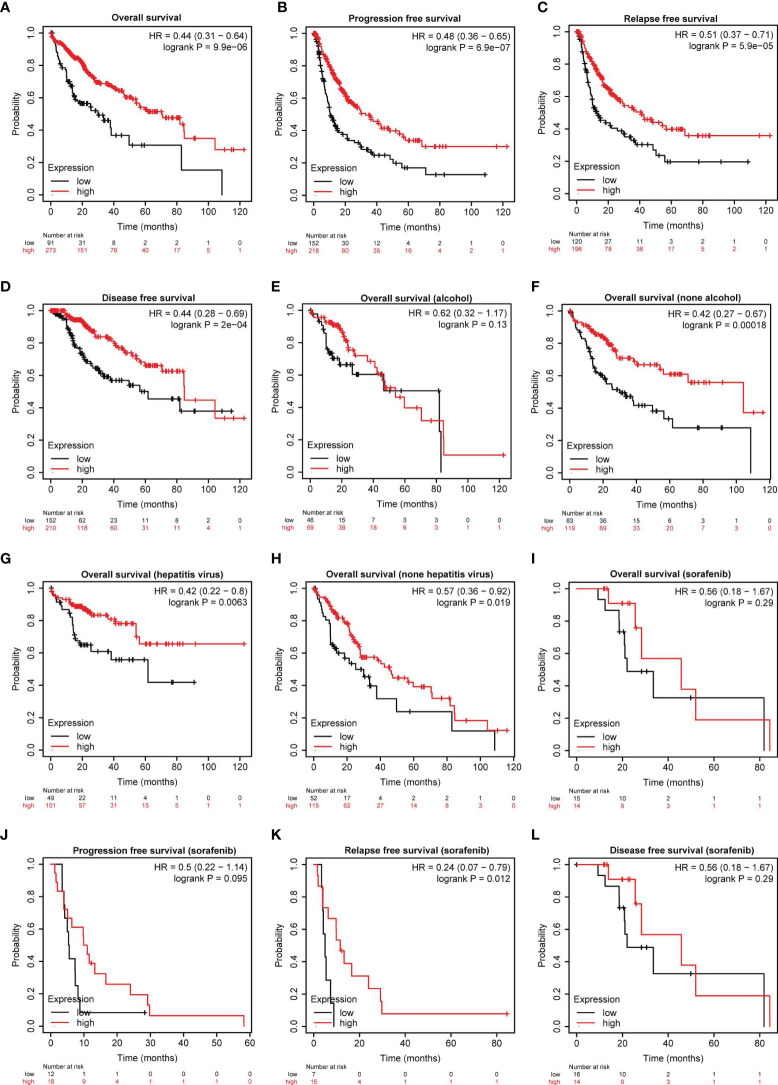
Serpinc1 protein expression is correlated with outcomes in hepatocellular carcinoma patients. **(A–D)** Analysis of the correlation between the serpinc1 expression and survival rate in hepatocellular carcinoma patients from Kaplan-Meier Plotter database. Overall survival **(A)**, Progression-free survival **(B)**, Relapse-Free survival **(C)**, and Disease-free survival **(D)**. **(E, F)** Analysis of the effect of alcohol and serpinc1 expression on the outcomes from Kaplan-Meier Plotter database. Alcohol **(E)** and none alcohol **(F)**. **(G, H)** Detection of the effect of Hepatitis virus and serpinc1 expression on the outcomes from Kaplan-Meier Plotter database. Hepatitis virus **(G)** and none hepatitis virus **(H)**. **(I–L)** Analysis of the effect of serpinc1 expression and sorafenib administration on outcomes of hepatocellular carcinoma patients from Kaplan-Meier Plotter database. Overall survival **(I)**, Progression-free survival **(J)**, Relapse-Free survival **(K)**, and Disease-free survival **(L)**.

To further evaluate the clinical significance of serpinc1, we used Kaplan-Meier plotter database (http://www.kmplot.com/analysis/index.php?p=background) to examine the relationship between serpinc1 expression and the outcomes of HCC patients (27). Serpinc1 expression was positively correlated with overall survival, progression-free survival, relapse-free survival, and disease-free survival of HCC patients (**Figures 2A–D**). As drinking alcohol and hepatitis virus infection are the main risk factors for hepatocellular carcinoma, we wanted to know whether these factors affect the effect of serpinc1 on the clinical outcome of HCC. HCC patients were divided into alcohol and none alcohol, or hepatitis virus and none hepatitis virus group. Serpinc1 expression was not correlated with overall survival in alcohol group (**Figure 2E**), while serpinc1 expression was still positively correlated with overall survival in none alcohol group (**Figure 2F**). Thus, drinking alcohol might disturb the role of serpinc1 in HCC. However, serpinc1 expression was positively correlated with overall survival in both hepatitis virus (**Figure 2G**) and none hepatitis virus (**Figure 2H**) groups, indicating that virus infection has no effect on the function of serpinc1.

In addition to risk factor analysis, we next studied the relationship between sorafenib (the major first-class drug for HCC treatment) treatment and serpinc1 expression on clinical outcomes in HCC. Serpinc1 was positively correlated with relapse-free survival in sorafenib group (**Figures 2I–L**). These findings further confirmed that serpinc1 is a biomarker of HCC and can predict the effect of chemotherapy.

### Serpinc1 Mediates Apoptosis in HCC Cells

**Figure 3:**
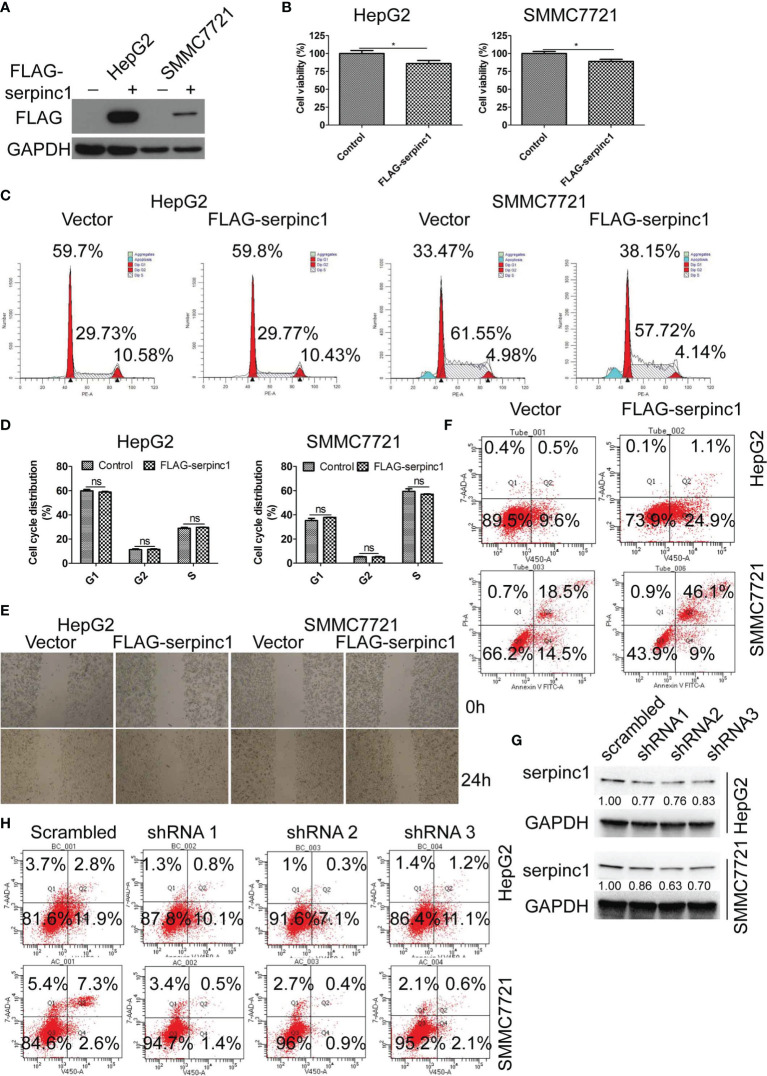
Serpinc1 overexpression induced apoptosis in HepG2 and SMMC7721 cells. **(A–F)** HepG2 and SMMC7721 cells were overexpressed with control or serpinc1. **(A)** Western blot to detect the expression level of serpinc1. **(B)** MTS to detect the cell viability. **(C, D)** Detection of cell cycle distribution. Representative images **(C)** and statistical analysis **(D)** of cell cycle distribution. **(E)** Wound healing assay to analyze the migration of hepatocellular carcinoma cell lines. **(F)** Flow cytometry to analyze the apoptosis. **(G, H)** Scrambled or serpinc1 shRNA were transfected in HepG2 and SMMC7721 cells. The knockdown of serpinc1 was detected by western blot **(G)**, and the cell apoptosis was detected by flow cytometry **(H)**. *P < 0.05, ns, no significant, *P* > 0.05.

To define a direct role of serpinc1 in HCC, we overexpressed serpinc1 in two HCC cell lines: HepG2 and SMMC7721 (**Figure 3A**). Cell viability assays showed that the overexpression of serpinc1 inhibited cell growth in HepG2 and SMMC7721 cells (**Figure 3B**), which was not associated with the change in cell cycle distribution (**Figures 3C, D**) and cell migration (**Figure 3E**). Further flow cytometry analysis revealed that the overexpression of serpinc1 induced apoptosis in HepG2 (from 10.1 to 26.0%) and SMMC7721 (from 33.0 to 55.1%) cells (**Figure 3F**). Serpinc1 overexpression repressed pro-survival-associated proteins BCL2, BCL-XL, and MCL-1 expression, while promoted apoptosis-related protein BAX expression (**Figure Supplemental 1A**). Accordingly, the knockdown of serpinc1 in HepG2 and SMMC7721 cells by three different shRNAs (**Figure 3G**) reduced the occurrence of apoptosis (**Figure 3H**). Meanwhile, serpinc1 knockdown induced BCL2, BCL-XL, MCL-1 instead of BAX (**Figure Supplemental 1B**). These findings suggested that serpinc1 is a positive regulator of apoptosis in HCC cells.

### Serpinc1 Inhibits M2 Polarization in Macrophages

**Figure 4:**
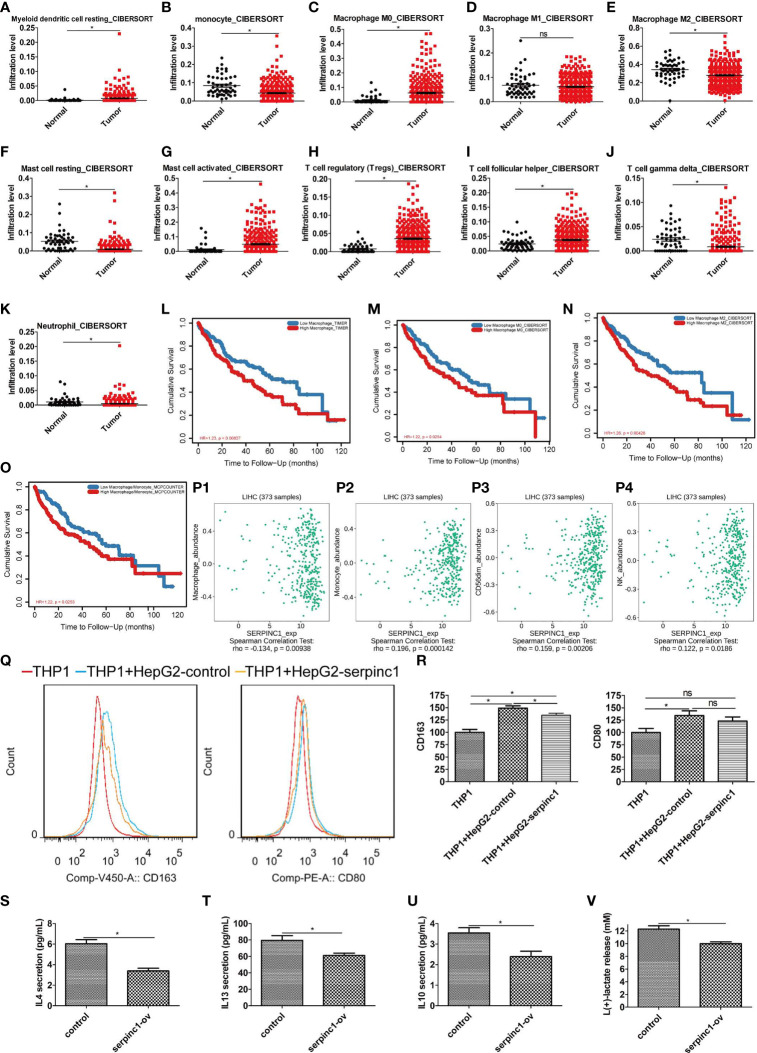
Serpinc1 expression inhibited macrophage M2 immune infiltration in hepatocellular carcinoma. **(A–K)** Analysis of immune cell infiltration abundance in hepatocellular carcinoma and normal controls with CIBERSORT. Myeloid dendritic cell resting **(A)**, Monocyte **(B)**, Macrophage M0 **(C)**, Macrophage M1 **(D)**, Macrophage M2 **(E)**, Mast cell resting **(F)**, Mast cell activated **(G)**, T cell regulatory **(H)**, T cell follicular helper **(I)**, T cell gamma delta **(J)**, and Neutrophil **(K)**. **(L–O)** Analysis of correlation between lymphocyte and outcomes in hepatocellular carcinoma patients from TIMER2.0 database. Macrophage **(L)**, macrophage 0 **(M)**, macrophage 2 **(N)**, and macrophage/monocyte **(O)**. **(P1–P4)** Analysis of correlation between serpinc1 and immune infiltration from TISIDB database. Macrophage **(P1)**, monocyte **(P2)**, NK CD56dim **(P3)**, and NK **(P4)**. **(Q, R)** THP1 cells activated by 50 nM PMA were co-cultured with HepG2 overexpressed control (blue) or serpinc1 (yellow) or alone (red). Macrophage M2 marker CD163 and M1 marker CD80 were detected by flow cytometry. Representative image **(Q)** and statistical analysis **(R)**. **(S–V)** HepG2 cells were overexpressed control or serpinc1, and analyzed the macrophage M2-associated factors in culture medium. Statistical analysis of IL4 **(S)**, IL13 **(T)**, IL 10 **(U)**, and lactate **(V)**. *P < 0.05, ns, no significant, *P* > 0.05.

To determine the status of tumor immunity in HCC, data from TCGA HCC was collected, and then the immune infiltration degrees were analyzed using CIBERSORT method (http://cibersort.stanford.edu/) (28) in TIMER (http://timer.cistrome.org/) (29). Compared to normal controls, myeloid dendritic cells were increased, while monocytes were decreased in HCC patients (**Figures 4A, B**). Since monocytes can differentiate into macrophages, we also detected infiltration of different types of macrophages. The number of macrophage M0 was increased, M2 was decreased, whereas M1 remained unchanged (**Figures 4C–E**). Of note, macrophage M2 was the most abundant immune infiltration cells in HCC. Resting mast cells were decreased (**Figure 4F**), while activated mast cells were increased (**Figure 4G**). The regulatory and follicular helper T cells were increased (**Figures 4H, I**), while gamma delta T cells and neutrophils were decreased in HCC (**Figures 4J, K**). These data indicate that monocytes are promoted to differentiate to macrophage M0, while the differentiation of macrophages M0 to M2 was blocked in HCC. Subsequent survival analysis found that the abundance of macrophages, macrophages M0, macrophages M2, and macrophages/monocytes was negatively correlated with the survival rate of HCC (**Figures 4L** **–O**). Therefore, the polarization state of macrophages is an important factor affecting the prognosis of HCC.

Next, we studied the role of serpinc1 in HCC immune cell regulation. We employed TISIDB database to analyze their relationships. Interestingly, serpinc1 expression was negatively correlated with macrophages in HCC (**Figure 4** **P1**). However, serpinc1 expression was positively correlated with monocytes, nature killer cells, and nature killer cells CD56_dim_ (**Figures 4P2–P4**).

To determine the direct role of serpinc1 in regulating the production of macrophage M2, we used a co-culture system containing THP1 cells (human monocytes) and HepG2 cells to assay the expression of macrophage M2 marker CD163 and M1 marker CD80. Compared to control HepG2 cells, serpinc1-overexpressed HepG2 cells inhibited the production of CD163 in THP1 cells. However, serpinc1-overexpressed HepG2 cells did not affect the production of CD80 in THP1 cells (**Figures 4Q, R**). Moreover, serpinc1-overexpressed HepG2 dampened the production of macrophage M2-associated factors, including IL4 (**Figure 4S**), IL13 (**Figure 4T**), IL10 (**Figure 4U**), and lactate (**Figure 4V**) (30). These findings demonstrated that increased serpinc1 expression in HCC cells impairs macrophage M2 polarization in tumor microenvironment.

### Serpinc1 Regulates the Expression of Immune Molecules in HCC

**Figure 5:**
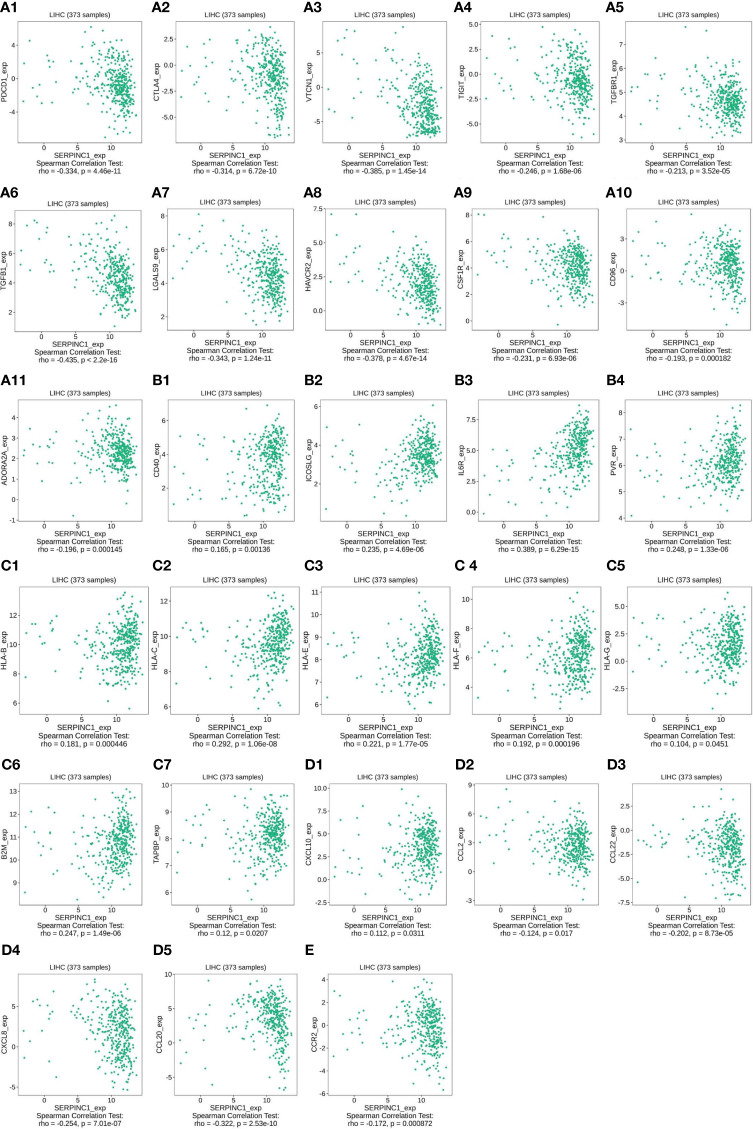
Serpinc1 expression regulated immune molecules in hepatocellular carcinoma. **(A1–A11)**, The relationship between immunoinhibitors and serpinc1 expression from TISIDB database. **(B1–B4)** The relationship between immunostimulators and serpinc1 expression from TISIDB database. **(C1–C7)** The relationship between MHC molecules and serpinc1 expression from TISIDB database. **(D1–D5)** The relationship between chemokines on trafficking of T cells to tumor or tumor-associated macrophage and serpinc1 expression from TISIDB database. **(E)** The relationship between receptor on tumor-associated macrophage and serpinc1 expression from TISIDB database.

Since serpinc1 can inhibit macrophage M2, we evaluated the relationship between serpinc1 expression and major immune molecules involved in tumor immunity in HCC through the TISIDB database. Serpinc1 expression was negatively correlated with immunoinhibitors, including PDCD1/PD-1 (**Figure 5A1**), CTLA4 (**Figure 5A2**), VTCN1 (**Figure 5A3**), TIGIT (**Figure 5A4**), TGFBR1 (**Figure 5A5**), TGFB1 (**Figure 5A6**), LGALS9 (**Figure 5A7**), HAVCR2 (**Figure 5A8**), CSF1R (**Figure 5A9**), CD96 (**Figure 5A10**), and ADORA2A (**Figure 5A11**). When serpinc1 was increased, the immunoinhibitors, especially the inhibitor of seventh step of cancer-immunity cycle, PDCD1, CTLA4, TGFB1 TGFBR1, and HAVCR2, were reduced. In contrast, serpinc1 expression was positively correlated with immunostimulators, such as CD40 (**Figure 5B1**), ICOSLG (**Figure 5B2**), IL6R (**Figure 5B3**), and PVR (**Figure 5B4**). Moreover, serpinc1 expression was positively correlated with MHC molecules, including HLA-B (**Figure 5C1**), HLA-C (**Figure 5C2**), HLA-E (**Figure 5C3**), HLA-F (**Figure 5C4**), HLA-G (**Figure 5C5**), B2M (**Figure 5C6**), and TAPBP (**Figure 5C7**). These assays suggested that serpinc1 promotes expression of MHCs, which mediated the presented antigens to T cells, the second step of cancer-immunity cycle.

Some chemokines, such as CXCL10, are important for the recruitment of effector T cells for antitumor immunity. Serpinc1 was positively correlated with CXCL10 (**Figure 5** **D1**) and negatively correlated with CCL2 (**Figure 5D2**), CCL22 (**Figure 5D3**), CXCL18 (**Figure 5D4**), and CCL20 (**Figure 5D5**) in HCC (**Figure 5D**). Finally, CCR2, the receptor on tumor-associated macrophage, was negatively correlated with serpinc1 expression in HCC (**Figure 5E**). Altogether, these data indicate that serpinc1 regulates the production of immune molecules in HCC.

### Serpinc1 Regulates Apoptosis and M2 Polarization *via* the UPS

**Figure 6:**
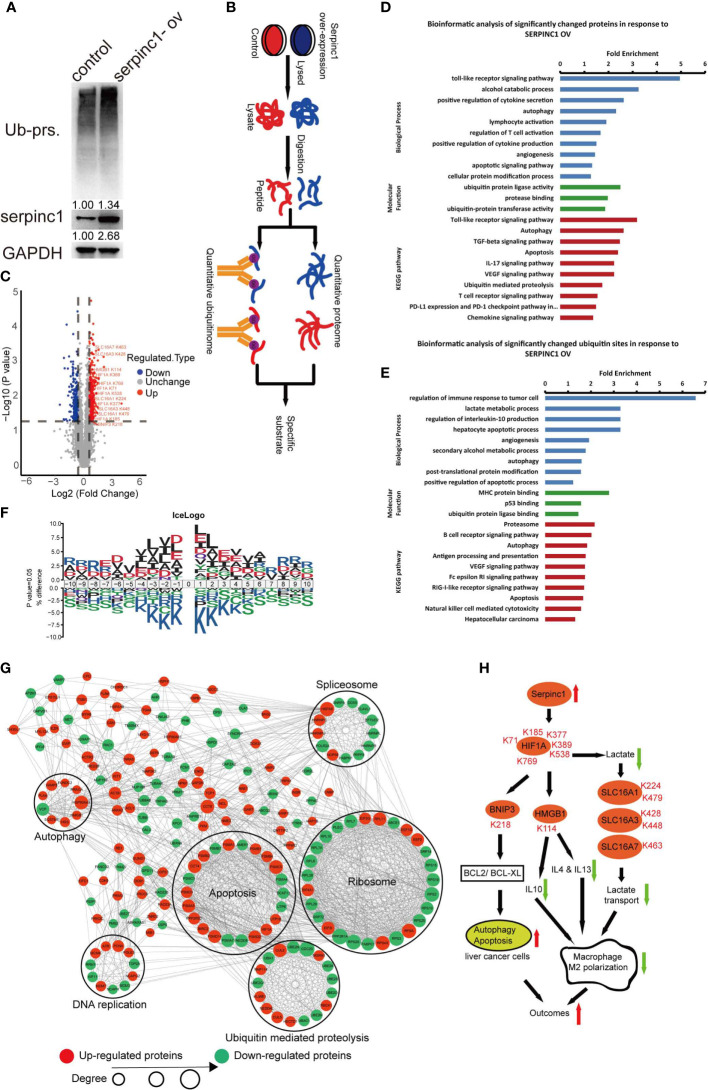
Serpinc1 overexpression orchestrated proteome and ubiquitinome in HepG2 cells. **(A)** Detection of ubiquitinated proteins, serpinc1 protein expression after overexpression of serpinc1 (serpinc1-ov) in hepatocellular carcinoma cell line HepG2 by western blot. **(B)** The diagram shows the experimental process of quantitative proteome and ubiquitinome of overexpression of serpinc1. **(C)** Volcano plot of ubiquitinated modified sites change in response to serpinc1 overexpression in HepG2 cells. **(D)** GO analysis and KEGG pathway of the significantly changed proteins identified in proteome. **(E)** GO analysis and KEGG pathway of the significantly changed ubiquitinated sites identified in ubiquitinome. **(F)** Motif enrichment of all ubiquitinated sites identified in ubiquitinome. **(G)** Protein-protein interaction of significantly changed ubiquitinated sites identified in ubiquitinome. **(H)** Scheme of serpinc1 regulated the apoptosis of liver cancer cells and macrophage M2 polarization.

To understand the mechanism by which serpinc1 regulates liver cancer cell apoptosis and macrophage M2 polarization, we wondered whether serpinc1 affects protein degradation due to its peptidase inhibitor properties. Indeed, the overexpression of serpinc1 led to the accumulation of ubiquitinated proteins in HepG2 cells (**Figure 6A**). Further quantitative proteome and ubiquitinome detailed serpinc1-related downstream substrates (**Figure 6B**). Serpinc1 overexpression resulted in an increase of 147 proteins and a decrease of 65 proteins by 1.5 folds (**Figure Supplemental 2A, B**). Quantitative ubiquitinome showed that the overexpression of serpinc1 upregulated 328 ubiquitinated sites in 260 proteins, while downregulated 244 ubiquitinated sites in 200 proteins, with a 1.5-fold change (**Figure 6C** and **Figure Supplemental 2C**). Of note, we also confirmed that HIF1A and HMGB1 ubiquitination were upregulated after serpinc1 overexpression *via* immunoprecipitation in HepG2 cells (**Figure Supplemental 1C, 1D**), which was consistent with LC-MS data.

Further GO and KEGG enrichment showed that the quantitative proteome was focused on autophagy, apoptosis, ubiquitin-proteasome, alcohol catabolism, and macrophage M2 polarization, such as cytokine secretion/production and VEGF signal pathways (**Figure 6D**). Moreover, quantitative ubiquitinome was enriched in autophagy, apoptosis, ubiquitin-proteasome, secondary alcohol metabolic process, and macrophage M2 polarization, such as regulation of interleukin-10 production, lactate metabolic process, VEGF signal pathway, and Fc epsilon RI signal pathway (**Figure 6E**). The GO enrichment including alcohol metabolism was consistent with **Figure 2E–F**, which supported that alcohol affects the relationship between serpinc1 expression and the prognosis of patients with HCC. The downstream pathway of serpinc1 also confirmed that serpinc1 regulated apoptosis and macrophage M2 polarization (**Figures 3F** and **Figures 4Q–R**).

To determine whether there are any amino acid biases surrounding the ubiquitinated sites, we analyzed the sequence commonalities of the detected ubiquitinated sites. The analysis resulted in seven motifs (KxL, DK, RxxxxxxxK, IxK, AK, KxxxxxxxR, and DxK), indicating that the fine amino acid residues, including L, D, R, I, and A, are overrepresented surrounding the lysine ubiquitinated sites (**Figure 6F**, **Figure Supplemental 2D, F**). Furthermore, the altered sites were enriched in pathways of ribosomes, apoptosis, ubiquitin-mediated proteolysis, spliceosomes, DNA replication, and autophagy (**Figure 6G**). The protein network also confirmed that serpinc1 mediated autophagy and apoptosis in HCC. Together, serpinc1 is an important regulator of the UPS, thereby affecting HCC biology.

## Discussion

The UPS is responsible for degrading proteins which are modified by ubiquitin catalyzed by ubiquitin activating enzymes (E1), ubiquitin conjugating enzymes (E2), and ubiquitin ligases (E3), while deubiquitinases oppose this process. Due to the increase in abnormal proteins, HCC cells have an altered UPS. In this study, we demonstrated that serpinc1 acts as a key regulator of the UPS, leading to HCC cell apoptosis and antitumor immunity (**Figure 6H**). These findings are consistent with previous preclinical and clinical studies that serpinc1 exerts a tumor suppressor effect in HCC.

First, we showed that serpinc1 mRNA and proteins increased in hepatocellular carcinoma, and this increase was negatively correlated with tumor grades. The increased serpinc1 expression was positively correlated with patient outcomes in HCC. In addition, drinking alcohol, rather than hepatitis virus infection, could block the positive correlation between serpinc1 expression and the prognosis of HCC patients. The quantitative proteome and ubiquitinome of serpinc1-overexpressed HCC cells also confirmed that serpinc1 can alter alcohol metabolism pathway. Thus, serpinc1 may function on HCC *via* alcohol catabolism, although the mechanism of upregulation of serpinc1 is unclear. In addition to risk factors, high serpinc1 expression predicted good prognosis (relapse-free survival) in HCC patients treated with first-class drug sorafenib. This suggests that upregulation of serpinc1 can predict drug responsiveness. Nevertheless, how the dysregulated serpinc1 signal affects sorafenib resistance is still a question for further study.

Second, we highlighted a function of serpinc1 to inhibit the growth of HCC cells by inducing apoptosis rather than triggering cell cycle arrest. Apoptosis is a highly regulated process that includes cell death receptors and mitochondrial pathways. In nasopharyngeal carcinoma cells, the knockdown of serpinc1 increases mitochondrial apoptosis through the upregulation of pro-apoptotic BAX and the downregulation of anti-apoptotic BCL2 and survivin (14). In contrast, we established the opposite effect of serpinc1 in promoting the apoptosis of HCC cells because it can change the ubiquitination sites of various proteins related to autophagy and apoptosis pathway. For example, the HIF1A/BNIP3 axis, the main autophagy and apoptosis regulator under hypoxia (31), was significantly upregulated at the ubiquitinated sites after serpinc1 is overexpressed. Further studies are warranted to confirm whether the impaired HIF1A/BNIP3 axis is the cause of serpinc1-induced apoptosis in HCC cells. Regardless, the cancer type-dependent role of serpinc1 in coordinating cell death and survival signals remains a challenge.

Third, we demonstrated a potential role of serpinc1 in inhibiting macrophage M2 polarization, thereby enhancing the antitumor immunity. The emerging tumor microenvironment is a complex system that includes cancer cells, immune cells, and other components. Generally, the hot tumor microenvironment may help immune cells activate to eliminate cancer cells. Unlike M1 macrophages that produce pro-inflammatory cytokines, macrophages M2 mainly cause immune paralysis or tolerance due to their production of anti-inflammatory cytokines. We showed that serpinc1-overexpressed HCC cells restrained macrophages M2 polarization. Our quantitative proteome and ubqiuitinome analysis further suggested that the HIF1A, an important transcription factor for immunometabolism, may be a key molecule for serpinc1-mediated suppression of M2 polarization. Alternatively, HMGB1 (a HIF1A downstream target and a redox mediator of immunogenic cell death) may participate in this process through IL 4, IL10, and IL13 (32–35). Lactate (another downstream signal of HIF1A) is transported through SLC16A1, SLC16A3, and SLC16A7, which is inhibited by the overexpression of serpinc1 (36–39). In addition to macrophage, the expression of serpinc1 also affects the number and types of other innate or adaptive immune cells in the HCC tumor microenvironment. It is important to further determine the role of serpinc1 in regulating immune cell communication and immune mediator production in HCC.

In summary, we found that upregulated serpinc1 is beneficial to inhibit HCC growth by inducing apoptosis and macrophage polarization. This process requires serpinc1-dependent regulation of the UPS. The identification of the substrate directly degraded by serpinc1 will help to further understand these regulatory mechanisms.

## Data Availability Statement

The mass spectrometry proteomics data have been deposited to the ProteomeXchange Consortium *via* the PRIDE (Perez-Riverol et al., 2019) partner repository with the dataset identifier PXD026782.

## Author Contributions

DX and JL designed the study. DX and JW conducted the apoptosis experiments. DX, LD, WL, and LL conducted macrophage polarization, interleukin secretion and lactate assays. DX analyzed data. DX and DT wrote the manuscript. All authors contributed to the article and approved the submitted version.

## Funding

The study was supported by the National Funds for Developing Local Colleges and Universities (B16056001), the Natural Science Foundation research team of Guangdong Province (2018B030312001), the Science and Technology Program of Guangzhou (201604020001), the Innovative Academic Team of Guangzhou Education System (1201610014), the Research Team of Department of Education of Guangdong Province (2017KCXTD027), the Post-doc initiation fund of Guangzhou (011302009), and the Post-doc science research initiation fund of Guangzhou Women and Children’s Medical Center (3001103).

## Conflict of Interest

The authors declare that the research was conducted in the absence of any commercial or financial relationships that could be construed as a potential conflict of interest.

## Publisher’s Note

All claims expressed in this article are solely those of the authors and do not necessarily represent those of their affiliated organizations, or those of the publisher, the editors and the reviewers. Any product that may be evaluated in this article, or claim that may be made by its manufacturer, is not guaranteed or endorsed by the publisher.
