# Supplementary material for: Serpinc1 Acts as a Tumor Suppressor in Hepatocellular Carcinoma Through Inducing Apoptosis and Blocking Macrophage Polarization in an Ubiquitin-Proteasome Manner
Source: Front Oncol. 2021 Nov 22;11:738607. doi: 10.3389/fonc.2021.738607 (PMC8645897; doi:10.3389/fonc.2021.738607)
Supplement: Supplementary file 1 [file DataSheet_1.pdf]

Supplementary Material

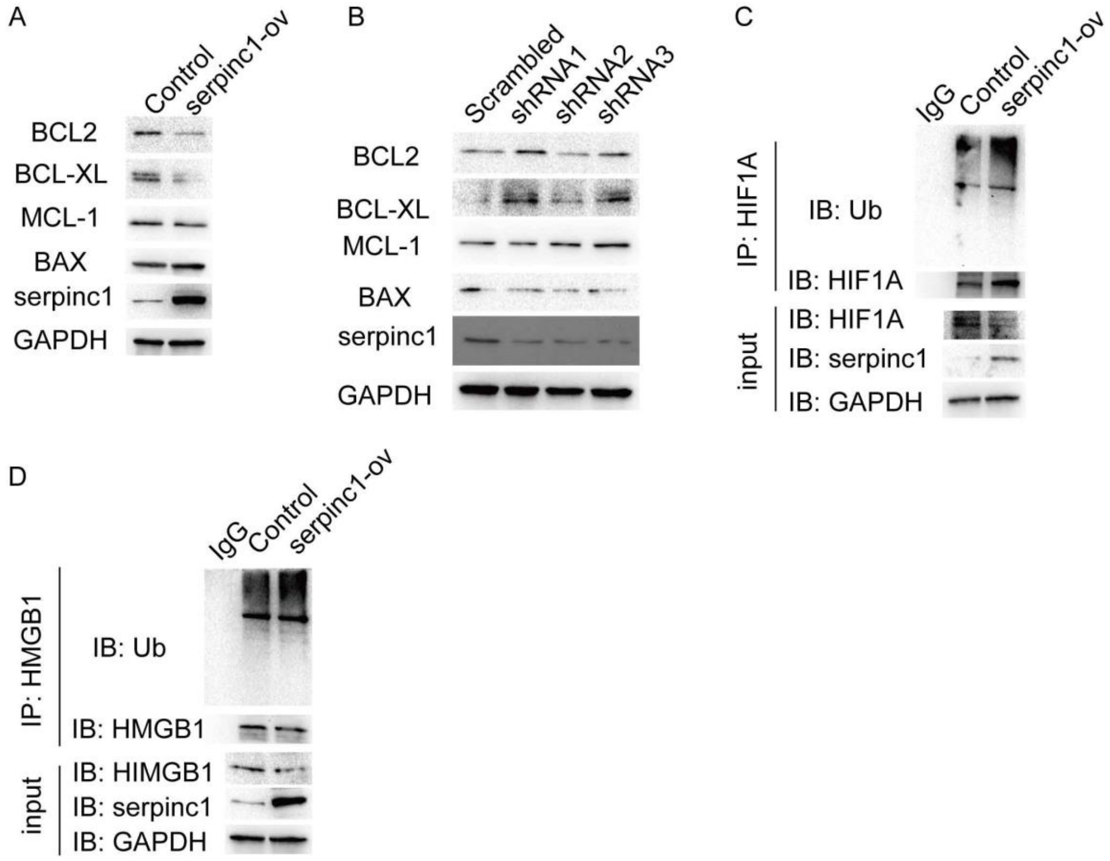

Figure supplemental 1. Serpinc1 regulated apoptosis protein expression and protein ubiquitination in HepG2 cells. A-B, serpinc1 gain-off effect on the pro-survival and apoptosis protein expression in HepG2 cells. Overexpression (A) or knockdown (B) of serpinc1. C-D, detection of serpinc1 overexpression effect on proteins ubiquitination in HepG2 cells. Ubiquitination of HIF1A (C) and HMGB1 (D).

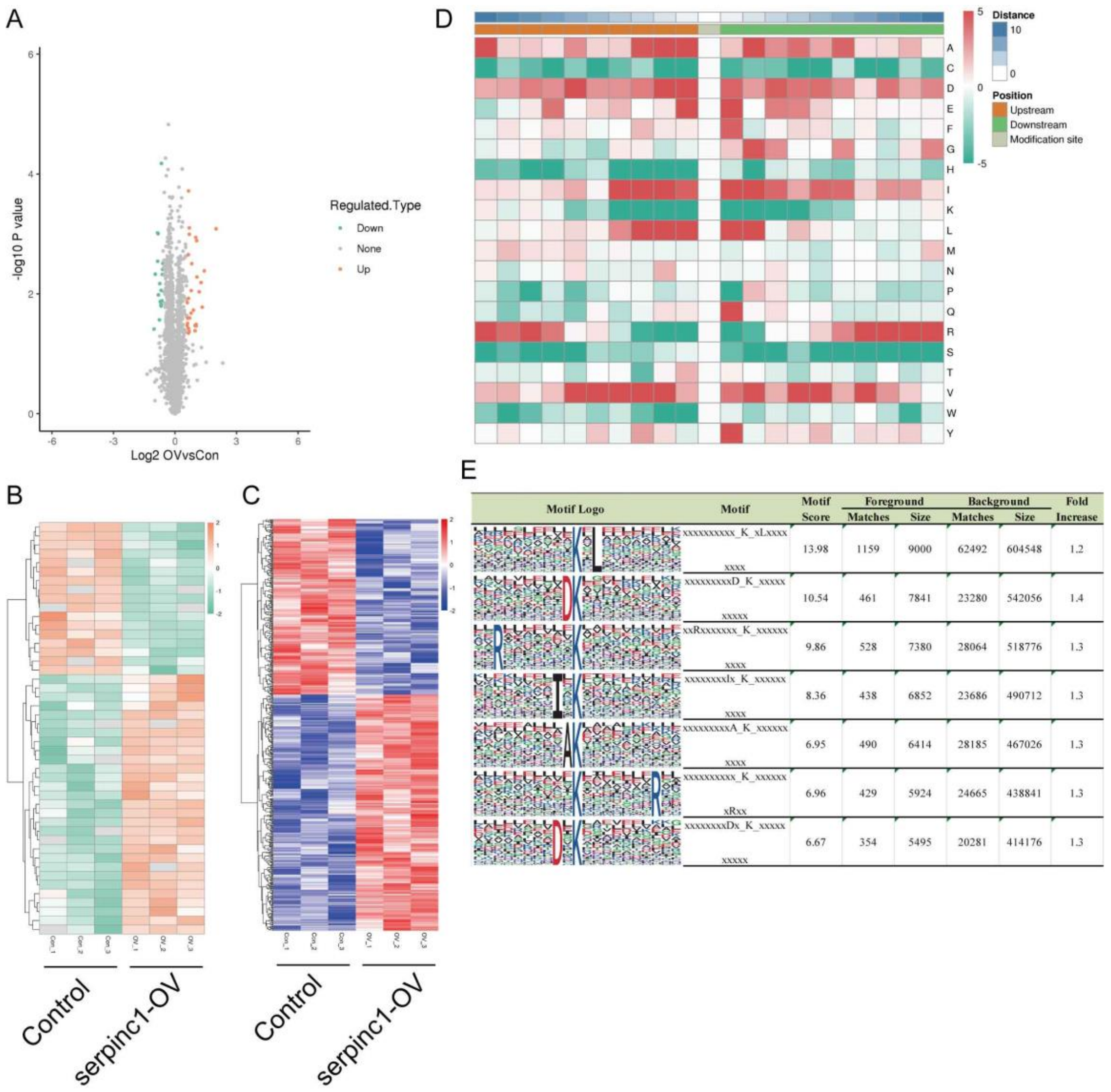

Motif Logo	Motif	Motif Score	Foreground Matches	Foreground Size	Background Matches	Background Size	Fold Increase
	xxxxxxxxxx_K_xLxxxx xxxx	13.98	1159	9000	62492	604548	1.2
	xxxxxxxxxD_K_xxxxx xxxxx	10.54	461	7841	23280	542056	1.4
	xxRxxxxxxx_K_xxxxxx xxxx	9.86	528	7380	28064	518776	1.3
	xxxxxxxxIx_K_xxxxxx xxxx	8.36	438	6852	23686	490712	1.3
	xxxxxxxxxA_K_xxxxxx xxxx	6.95	490	6414	28185	467026	1.3
	xxxxxxxxxx_K_xxxxxx xRxx	6.96	429	5924	24665	438841	1.3
	xxxxxxxxDx_K_xxxxx xxxxx	6.67	354	5495	20281	414176	1.3

Figure supplemental 2. Proteome and ubiquitinome of serpinc1 overexpression in HepG2 cells. A-B, Volcano plot (A) and heatmap (B) of significantly changed proteins in serpinc1 overexpression. C, heatmap of significantly altered ubiquitinated sites in serpinc1 overexpression. D-E, sequence commonalities (D) and motif (E) of ubiquitinated sites.
